# Intracellular *S. aureus* in Osteoblasts in a Clinical Sample from a Patient with Chronic Osteomyelitis—A Case Report

**DOI:** 10.3390/pathogens10081064

**Published:** 2021-08-22

**Authors:** Nike Walter, Daniel Mendelsohn, Christoph Brochhausen, Markus Rupp, Volker Alt

**Affiliations:** 1 Department for Trauma Surgery, University Hospital Regensburg, 93053 Regensburg, Germany; nike.walter@ukr.de (N.W.); Daniel.Mendelsohn@stud.uni-regensburg.de (D.M.); markus.rupp@ukr.de (M.R.); 2 Institute of Pathology, University Hospital Regensburg, 93053 Regensburg, Germany; christoph.brochhausen@ukr.de

**Keywords:** osteomyelitis, intracellular *S. aureus*, osteoblast, ultrastructural analysis

## Abstract

The pathophysiological role of intracellular bacteria in osteomyelitis is still a matter of debate. Here, we demonstrate for the first time the presence of *Staphylococcus aureus* internalized into osteoblasts in human tissue samples of a case with a chronic osteomyelitis using ultrastructural transmission electron microscope analysis.

## 1. Introduction

Osteomyelitis has affected humans from the very beginning. It still remains a difficult challenge in orthopaedic and trauma surgery with an incidence of 16.7 cases per 100,000 inhabitants in Germany [1]. Gram-positive pathogens such as *Staphylococcus aureus* are, in most cases, the cause of bone infections [2]. The biofilm theory introduced by Costerton has been widely accepted as a hallmark in the pathophysiology of osteomyelitis [3]. Another pathophysiological explanation, especially in light of the complexity of treating osteomyelitis, is the invasion of *S. aureus* into osteoblasts, which is still controversially discussed. For *S. aureus*, the invasion of osteoblasts has been demonstrated where this bacterium enters a ‘persister’ state, in which exposure to high levels of antibiotics can be survived due to a lack of metabolic activity. The internalization of *S. aureus* into osteoblasts has been shown in several in vitro studies [4,5]. However, to the best of our knowledge, the presence of intracellular *S. aureus* in osteoblasts in human tissue samples has not yet been evidenced.

## 2. Case Presentation

**Figure 1:**
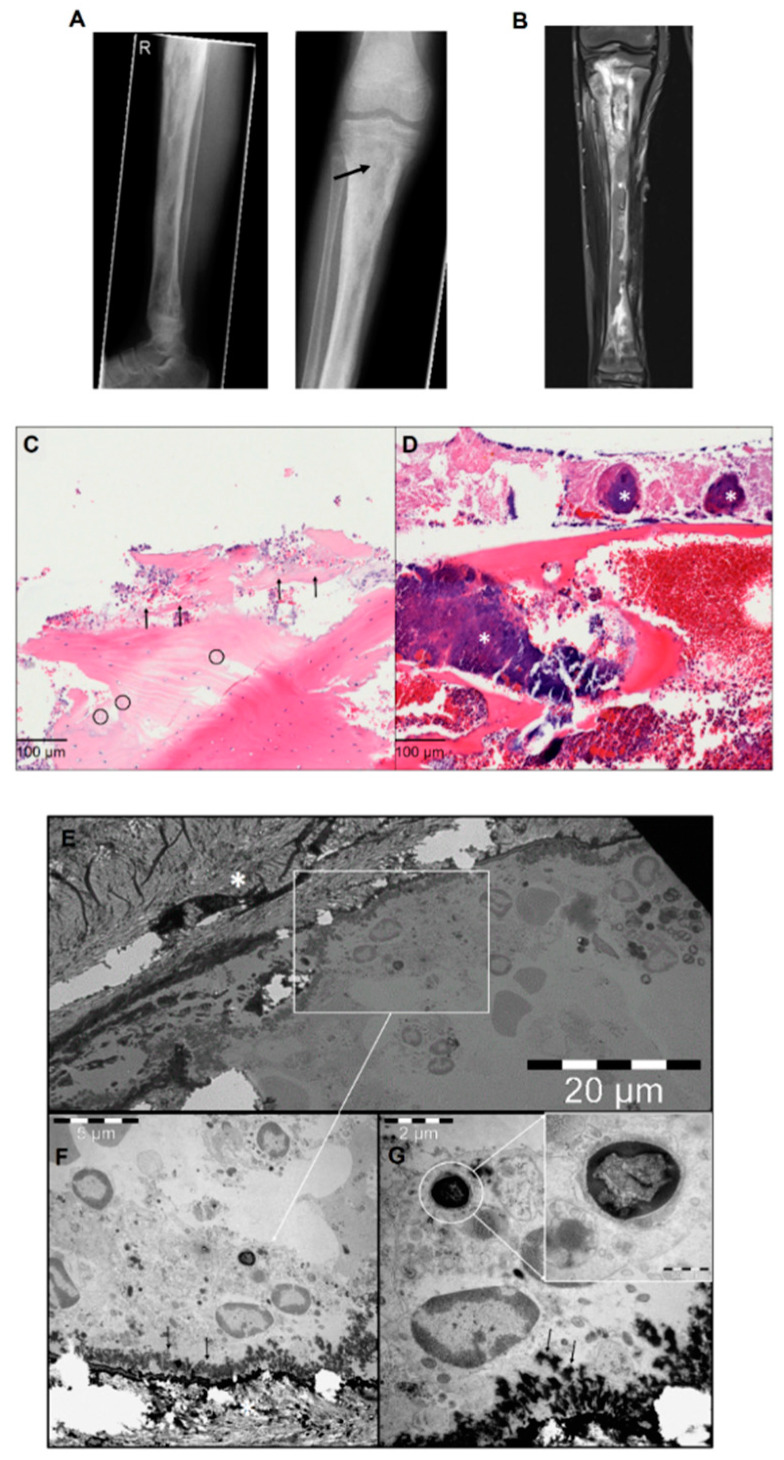
(**A**) Preoperative X-rays of the right tibia: periosteal reactions, cortical erosions, focal osteopenia, osteolysis and endosteal scalloping are detectable throughout the long bone. The black arrow in the right panel shows the sampling point. (**B**) Preoperative magnetic resonance imaging of the right tibia: signs of osteomyelitis are visible. (**C**) Histological findings of chronic osteomyelitis: bone necrosis with acute inflammatory infiltration (arrow) and empty osteocytic lacunae (circles). (**D**) Bone necrosis with high amounts of bacterial colonies (*) (HE, ×400). (**E**) Transmission electron microscope overview images of bone trabecula (marked with the white star) with adjacent osteoblasts. The white square marks the identified region of interest. (**F**) High magnification of osteoblasts with newly formed osteoid (black arrows) and intracellular *Staphylococcus aureus.* (**G**) Osteoblast with magnified intracellular *Staphylococcus aureus*.

We report the case of a 14-year-old boy with 10 months’ history of chronic osteomyelitis. He presented with secretion from multiple draining sinuses on the right leg. Laboratory analysis revealed a C-reactive protein level (CRP) of 63.2 mg/L (reference value: <5 mg/L) and a leucocyte count of 11.82/nL (reference value: 4.2–12.0/nL). Radiographs and MRI imaging of the leg showed typical features such as intramedullary abscess formation, cortical erosions, osteolysis and endosteal scalloping consistent with the diagnosis of chronic osteomyelitis of the entire tibia including the proximal and distal epiphysis (Figure 1A,B). During the first surgical intervention, performing thorough debridement and necrosectomy, eight tissue samples were harvested for microbiological and histological examination. Methicillin-resistant *S. aureus* was identified in all samples by the matrix-assisted laser desorption ionization time of flight mass spectrometry (MALDI TOF MS), using a Microflex LT mass spectrometer and BioTyper software (Bruker Daltonik, Bremen, Bremen Germany). Accordingly, results of the histological examination showed infection signs (Figure 1C,D). Further, parts of the same bone samples harvested during surgery underwent ultrastructural analysis. For this purpose, the samples were fixed with a Karnovsky-fixative (0.1 M cacodylate-buffer with 2.5% glutaraldehyde and 2% paraformaldehyde) followed by 1% of osmium tetroxide at pH 7.3. Next, the samples were decalcified and embedded in EMbed-812 epoxy resin (Science Services, Munich, Bavaria, Germany). After performing semi-thin sections of 0.75 μm thickness, sections were stained with toluidine blue and basic fuchsine solution and visualized using a light microscope to identify the specific regions of interest for further ultrastructural investigation. Finally, the sections were analyzed with a LEO912AB transmission electron microscope (Zeiss, Oberkochen, Baden-Württemberg, Germany) operating at 100 kV. High-resolution images were acquired with an integrated Sharp Eye 2k slow-scan CCD camera (TRS, Moorenweis, Bavaria, Germany). Ultrastructural analysis revealed the presence of bacteria within the vacuoles in osteoblasts, which in light of the consistent microbiological finding in all eight samples, can be deemed as *S. aureus* (Figure 1E–G).

## 3. Discussion

The presented case report confirms the internalization of *S. aureus* into human osteoblasts in vivo. So far, the presence of internalized bacteria in osteoblasts has been demonstrated by multiple in vitro studies [4,6,7,8,9,10,11] and a few in vivo studies utilizing chicken embryos [12]. However, to the best of our knowledge, the presence of intracellular *S. aureus* in patient samples has not yet been evidenced. Hitherto, Bosse and colleagues were the only authors to report a case of a 73-year-old patient with recurrent, long-term osteomyelitis showing intracellular *Streptococcus constellatus* in osteoblasts [13]. One reason for the novelty of our finding might be the difficulty in detecting viable cells in the bone, which is often necrotic in chronic osteomyelitis cases. The internalization of *S. aureus* might fundamentally contribute to the obstacles in the treatment of osteomyelitis. However, the scarcity of clinical data challenges the clinical relevance of a variety of in vitro studies devoted to unravel the mechanisms that govern osteomyelitis. Despite advances in pathophysiological understanding, the standards of treatment care have not changed essentially in recent years. Hence, further studies investigating the occurrence of *S. aureus* in osteoblasts in human samples depending on the duration of symptom onset are required. In our case, adequate debridement, and a local and empirical antibiotic therapy with a free microvascular latissimus dorsi flap for soft tissue reconstruction, resulted in the successful eradication of the infection, despite evidence of internalized *S. aureus*.

In conclusion, our findings revealed the presence of intracellular *S. aureus* in osteoblasts in clinical tissue samples from a patient with chronic osteomyelitis for the first time. This result should encourage researchers to further investigate the pathophysiology of osteomyelitis with special regard on the relevance of intracellular bacteria in osteoblasts and bacterial–cellular crosstalk.

## Data Availability

The datasets generated and analyzed in the current study are available from the corresponding author on reasonable request.
